# Preoperative thyroid hormone levels predict ICU mortality after cardiopulmonary bypass in congenital heart disease patients younger than 3 months old

**DOI:** 10.1186/s12887-021-02513-6

**Published:** 2021-01-25

**Authors:** Di Yu, Liang Zou, Yueshuang Cun, Yaping Li, Qingfeng Wang, Yaqin Shu, Xuming Mo

**Affiliations:** Department of Cardiothoracic Surgery, Children’s Hospital of Nanjing Medical University, Jiangdong South No. 8 Road, 210008 Nanjing, China

**Keywords:** Thyroid hormone, Congenital heart disease, Mortality

## Abstract

**Background:**

We aimed to study the effectiveness of preoperative thyroid hormone levels in predicting intensive care unit (ICU) mortality after cardiopulmonary bypass (CPB) in infants with congenital heart disease (CHD).

**Methods:**

We retrospectively reviewed and analyzed data from 133 patients younger than 3 months old who underwent cardiac surgery with CPB from June 2017 to November 2019. ICU mortality prediction was assessed by multivariate binary logistic regression analysis and area under the curve (AUC) analysis.

**Results:**

Non-survivors were younger (17.46 ± 17.10 days vs. 38.63 ± 26.87 days, *P* = 0.006), with a higher proportion of neonates (9/13 vs. 41/120, *P* = 0.017) and a higher proportion of individuals with a Risk Adjustment for Congenital Heart Surgery-1 (RACHS-1) score ≥ 4 (8/13 vs. 31/120, *P* = 0.020). No significant difference was found in CPB and aortic cross-clamping (ACC) time. The levels of free triiodothyronine (FT3) (3.91 ± 0.99 pmol/L vs. 5.11 ± 1.55 pmol/L, *P* = 0.007) and total triiodothyronine (TT3) (1.55 ± 0.35 nmol/L vs. 1.90 ± 0.57 nmol/L, *P* = 0.032) were higher in survivors than in non-survivors. In the ICU mortality prediction assessment, FT3 was an independent mortality predictor and showed a high AUC (0.856 ± 0.040).

**Conclusions:**

The preoperative FT3 level was a powerful and independent predictor of ICU mortality after CPB in infants with CHD younger than 3 months old.

## Background

Congenital heart disease (CHD) is the most common congenital malformation among live births, accounting for approximately one-third of birth defects [1]. A systematic review showed that the prevalence of CHD increased from 0.6 ‰ in the 1930s to 9.1 ‰ at the end of the 20th century [2]. Cardiopulmonary bypass (CPB) is often used for surgery in complex CHD cases. However, hemodilution, hypothermia and ultrafiltration during CPB have been shown to induce a temporary hypothyroid state, especially in infants [3, 4].

The thyroid hormones triiodothyronine (T3) and thyroxin (T4) have permissive effects on β1-adrenergic receptors, which enhance heart contractility and reduce systemic vascular resistance [5, 6]. Additionally, thyroid hormones increase preload and decrease afterload, leading to increased cardiac output [7]. Several studies have shown that the hypothyroid state, which affects the myocardial energy metabolism, is associated with poor prognosis after cardiac surgery with CPB [8, 9]. Correspondingly, thyroid hormone replacement therapy could provide clinical benefits in infants undergoing CPB [10–12].

A study was conducted to assess the effects of CPB on thyroid function in infants weighing less than 5 kg, and the results showed that low T3 and T4 levels were both predictors of high mortality [13]. Talwar et al. [14] found that low postoperative total T4 (TT4) levels were correlated with postoperative morbidity, a prolonged postoperative course, and prolonged mechanical ventilation (MV) in open heart surgery with CPB. Since thyroid hormone levels play a critical role in recovery from cardiac surgery and thyroid hormones decrease after CPB, the preoperative level of thyroid hormones could be a predictor of intensive care unit (ICU) mortality after CPB in CHD patients. Therefore, we conducted a retrospective study to evaluate the effect of preoperative thyroid hormone levels in relation to survival in patients after cardiac surgery with CPB.

## Methods

We retrospectively reviewed the medical records of patients with CHD younger than 3 months old in our hospital between June 2017 and November 2019. This study protocol was approved by the Institutional Ethical Committees of the hospital (201912257-1). We excluded patients who were older than 90 days at the time of surgery, patients without CPB, patients with primary thyroid gland disease, and patients with trisomy 21 syndrome. Clinical data included gender; age; weight; Risk Adjustment for Congenital Heart Surgery-1 (RACHS-1) score [15]; type of CHD; preoperative serum albumin level (normal range: 40–55 g/L); preoperative thyroid hormone levels [total T3 (TT3, normal range: 1.29–3.11 nmol/L), free T3 (FT3, normal range: 2.8–7.1 pmol/L), TT4 (normal range: 66-187.4 nmol/L), free T4 (FT4, normal range: 12.1–22 pmol/L), thyroid stimulating hormone (TSH, normal range: 0.2-5 µIU/ml)], which is a routine examination for patients with CHD in our clinic; CPB time; aortic cross-clamping (ACC) time; and ICU mortality.

### Statistical analysis

The statistical analysis was performed with SPSS version 20.0 software (Chicago, IL, USA). Continuous variables are expressed as the mean ± standard deviation, while categorical variables are summarized as frequencies and percentages. Comparisons between groups were performed using an unpaired Student’s t-test for continuous variables and a χ^2^ or Fisher’s exact test for categorical variables. Multivariate binary logistic regression analysis was further conducted to assess the independent ICU mortality predictors. Receiver operating characteristic (ROC) curves were generated to examine the ability of variables to predict ICU mortality, and the area under the curve (AUC) was calculated from the ROC curve. Youden’s index, which maximizes the sum of the sensitivity and specificity, was used to define the optimal cut-off value. Statistical significance was defined as *P* < 0.05.

## Results

### Patient characteristics

This study enrolled 133 patients younger than 3 months old (with a mean age of 36.56 ± 26.78 days), including 50 neonates (37.6 %). Eleven patients were premature, and their age was adjusted according to their gestational age. Among the participants, 39 patients (29.3 %) had a RACHS-1 operative risk score ≥ 4. Based on the normal ranges mentioned above, 10(7.5 %), 1(0.8 %), 23(17.3 %), 2(1.5 %) and 48(36.1 %) patients had low FT3, low FT4, low TT3, low TT4 and high TSH levels, respectively. A total of 13 patients (9.8 %) died in the ICU; their causes of death were low cardiac output syndrome (8/133, 6.0 %), sepsis (3/133, 2.3 %), and brain injury (2/133, 1.5 %). The demographic and physiological characteristics of the patients are presented in Table 1.

**Table 1:** Characteristics of enrolled patients with congenital heart disease

Characteristic	All patients (n = 133)
**Age (days)**	36.56 ± 26.78
≤28 days	50 (37.6 %)
>28 days and ≤ 90 days	83 (62.4 %)
**Gender**	
Male	89 (66.9 %)
Female	44 (33.1 %)
**Weight (kg)**	3.89 ± 0.91
**RACHS-1**	
Score-1	4 (3.0 %)
Score-2	46 (34.6 %)
Score-3	44 (33.1 %)
Score-4	38 (28.6 %)
Score-5	1 (0.7 %)
Score-6	0(0 %)
**Type of Surgery**	
ASD	2 (1.5 %)
VSD	12 (9.0 %)
VSD + ASD	50 (37.6 %)
COA/IAA	15(11.3 %)
TAPVC	21(15.8 %)
TGA	19 (14.3 %)
PA-VSD	3 (2.2 %)
DORV	2 (1.5 %)
Other	9 (6.8 %)
**Operative factors**	
CPB time (min)	114.23 ± 81.98
ACC time (min)	46.03 ± 21.59
**ICU mortality**	13 (9.8 %)
**Preoperative serum albumin (g/L)**	38.23 ± 4.87
**Preoperative thyroid function**	
FT3 (pmol/L)	4.99 ± 1.54
FT4 (pmol/L)	21.28 ± 5.82
TT3 (nmol/L)	1.87 ± 0.56
TT4 (nmol/L)	120.25 ± 34.61
TSH (µIU/ml)	5.72 ± 5.95

*RACHS-1* Risk Adjustment in Congenital Heart Surgery-1, *ASD* atrial septal defect, *VSD* ventricular septal defect, *COA* coarctation of aorta, *IAA* interrupter aortic arch, *TAPVC* total anomalous pulmonary venous connection, *TGA* transposition of the great arteries, *PA* pulmonary atresia, *DORV* double outlet right ventricular, *CPB* cardiopulmonary bypass, *ACC* aortic cross-clamping, *FT3* free triiodothyronine, *FT4* free thyroxin, *TT3* total triiodothyronine, *TT4* total thyroxin, *TSH* thyroid stimulating hormone

### Comparison of the survivor and non-survivor groups

Compared with survivors, non-survivors were younger (17.46 ± 17.10 days vs. 38.63 ± 26.87 days, *P* = 0.006), were more likely to be neonates (9/13 vs. 41/120, *P* = 0.017), and had a higher proportion of RACHS-1 scores ≥ 4 (8/13 vs. 31/120, *P* = 0.020), but there was no significant difference in preoperative serum albumin levels. The CPB and ACC time were slightly longer in the non-survivors, but no significant difference was found compared with the survivors. The non-survivors had low levels of FT3, FT4, TT3 and TT4 and a high TSH; however, the FT3 (3.91 ± 0.99 pmol/L vs. 5.11 ± 1.55 pmol/L, *P* = 0.007) and TT3 (1.55 ± 0.35 nmol/L vs. 1.90 ± 0.57 nmol/L, *P* = 0.032) levels showed a significant difference between survivors and non-survivors. Interestingly, all non-survivors were male (Table 2).

**Table 2:** Characteristics of patients according to survivors and non-survivors

Characteristic	Survivors (*N* = 120)	Non-survivors (*N* = 13)	*P* value
**Age (days)**	38.63 ± 26.87	17.46 ± 17.10	**0.006**
≤28 days	41 (34.1 %)	9(69.2)	**0.017**
**Female**	44 (36.7 %)	0 (0 %)	**0.005**
**Weight (kg)**	3.92 ± 0.93	3.61 ± 0.63	0.234
**RACHS-1 ≥ 4**	31(25.8 %)	8(61.5 %)	**0.020**
**Preoperative serum albumin (g/L)**	38.13 ± 4.97	39.31 ± 3.78	0.406
**Preoperative thyroid function**			
FT3 (pmol/L)	5.11 ± 1.55	3.91 ± 0.99	**0.007**
FT4 (pmol/L)	21.52 ± 5.79	19.02 ± 5.85	0.142
TT3 (nmol/L)	1.90 ± 0.57	1.55 ± 0.35	**0.032**
TT4 (nmol/L)	122.07 ± 34.77	103.47 ± 29.10	0.066
TSH (µIU/ml)	5.48 ± 4.94	7.89 ± 11.90	0.483
**Operative factors**			
CPB time (min)	111.53 ± 83.87	139.07 ± 58.61	0.251
ACC time (min)	45.01 ± 21.61	55.40 ± 19.73	0.100

Abbreviations as in Table 1

### Independent predictors of ICU mortality

Predictors with a P-value less than 0.1, except gender, were included in the multivariate binary logistic regression analysis to determine the independent predictors of ICU mortality. As shown in Table 3, FT3 was an independent predictor of mortality.

**Table 3:** Multivariate logistic regression, odds ratio of variables for predicting ICU mortality in patients with congenital heart disease after cardiopulmonary bypass

Variables	B	S.E.	Wald	df	*P* value	OR	95 % C.I. for OR	
							Lower	Upper
Age	-0.036	0.016	3.406	1	0.065	0.965	0.929	1.002
RASCH-1 ≥ 4	-1.002	0.528	1.621	1	0.203	0.367	0.078	1.717
ACC	-0.003	0.016	0.038	1	0.846	0.997	0.966	1.028
FT3	-1.112	0.528	4.429	1	**0.035**	**0.329**	**0.117**	**0.926**
TT3	2.227	1.437	2.400	1	0.121	9.268	0.554	155.017
TT4	-0.017	0.012	2.121	1	0.145	0.983	0.960	1.006
Constant	2.560	1.693	2.286	1	0.131	12.937		

Abbreviations as in Table 1

### Value of FT3 in predicting ICU morality

**Fig. 1:**
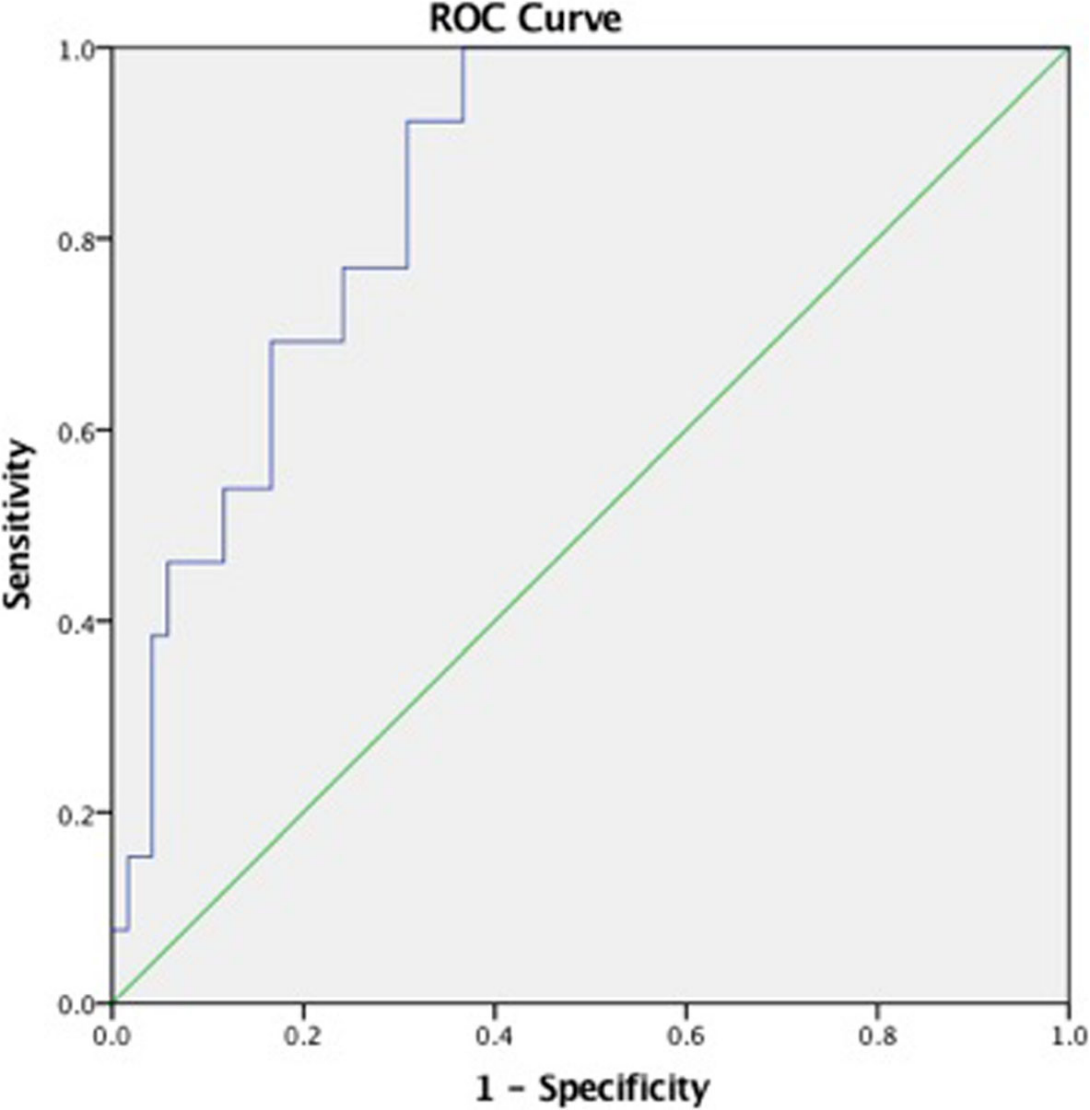
Receiver operating characteristic curves for free triiodothyronine (FT3)

ROC curves were constructed to examine the performance of FT3 as a predictor of ICU mortality (Fig. 1). The AUC was 0.856 ± 0.040, the optimal cutoff value was 4.89 pmol/L, and the sensitivity and specificity were 100 % and 63.3 %, respectively (Table 4).

**Table 4:** Value of FT3 in predicting ICU morality

Variable	AUC	P valve	Cutoff value	Sensitivity (%)	Specificity (%)
FT3	0.856 ± 0.040	0.000	4.89	100	63.3

*FT3* free triiodothyronine, *AUC* area under the curve

## Discussion

To the best of our knowledge, the present study is the first clinical retrospective analysis of the predictive value of preoperative thyroid hormone levels in patients with CHD undergoing CPB. In our study of 133 consecutive patients, we found that FT3 may be an independent predictor of ICU mortality based on multivariate binary logistic regression and ROC curve analyses. Previous studies [16] have reported that a low T3 level was an independent predictor of ICU mortality, which is consistent with our finding. However, for CHD patients, especially children, RACHS-1 scores could predict ICU mortality, length of ICU stay and duration of MV [17–20]. In our study, compared with survivors, non-survivors had higher RACHS-1 scores. However, in the additional multivariate binary logistic regression analysis, the RACHS-1 score was not an independent mortality predictor. We suggest that RACHS-1 scores on the basis of CHD subtype should not consider the relationship of year, weight, and levels of thyroid hormone as confounding factors [21]. Additionally, the small sample size of children with RACHS-1 scores of 5 or 6 may be another reason for the reduced statistical power. Although the CPB and ACC time were high in non-survivors, no significant difference was found compared with survivors, which demonstrates the improvement of cardiac surgery techniques and perfusion mode of CPB in China.

We found an interesting phenomenon in our study, namely, that all of the non-survivors were male. This could be related to the preference for sons over daughters, which is very common in China. Thus, when a child is diagnosed with complex CHD, based on the gender, operative risk and economic status of the parents, girls may not have the opportunity to undergo the operation, especially in rural areas [22, 23]. However, gender showed significant association with ICU mortality, but considering the gender selective bias, we removed gender from the multivariate binary logistic regression.

Thyroid hormones have important effects on the cardiovascular system, such as increasing cardiac output and decreasing systemic vascular resistance, which are predictive of good outcomes [24, 25]. However, several studies have verified that cardiac surgery with CPB induces a marked depression of thyroid hormones [14, 26, 27]. Researchers have found that low T3 [28] or T4 [14] levels were correlated with postoperative morbidity in open heart surgery with CPB. A study was conducted to assess the effects of CPB on thyroid function in infants weighing less than 5 kg, and the results showed that low T3 and T4 levels were both predictors of high mortality [13]. Since low postoperative low levels of thyroid hormones could lead to a poor prognosis, a preoperative increase in thyroid hormones may improve the prognosis. A multicenter randomized controlled trial (RCT) of T3 supplementation of patients undergoing heart surgery with CPB (TRICC) showed that T3 supplementation provides clinical advantages in patients younger than 5 months, but not in older patients [29]. Talwar et al. [10] performed an RCT study of perioperative oral T4 in patients younger than 6 months who underwent open heart surgery with CPB and found that postoperative thyroid hormone levels were reduced and that T4 supplementation reduced the duration of MV and ICU and hospital stays. Therefore, the preoperative level of thyroid hormones may predict the prognosis of patients with CHD undergoing CPB. Kumar et al. [30] found that low T3 is an important marker of mortality in critically ill patients, while low T4 and TSH levels did not increase the predictability of mortality. A large-scale prospective, observational study of unselected ICU patients, found that FT3 was the most powerful and independent predictor of ICU mortality among the thyroid hormone indicators [16]. However, Quispe et al. [31] found that the FT3 level was not significantly different between survivors and non-survivors and was not a mortality predictor. This might have occurred because previous studies did not consider the relationship of FT3 with albumin as confounding factor, and when hypoalbuminemia was present, conversion of T4 to T3 was decreased, resulting the low FT3 levels [32]. However, there was no significant difference in the preoperative albumin level between survivors and non-survivors in our study. Additionally, acute critically ill patients release large mounts of other hormones, such as cortisol, which has an inhibitory effect on TSH and eventually leads to low T3 levels [33]. In our study, patients were not in stress states and had similar preoperative albumin levels, and with the exclusion of these two confounding factors, we found that the preoperative FT3 level may be a predictor of ICU mortality after CPB in infants with CHD younger than 3 months old.

Our study showed that FT3 is an independent predictor of ICU mortality because of its high AUC value. Thyroid hormones include T4, which represents the major form of circulating thyroid hormones (> 80 %), and T3, which accounts for a small portion (< 20 %) of circulating thyroid hormone and has a major biological effect on the heart. Moreover, the levels of TT3 and TT4 levels can be affected by the thyroxine-binding globulin (TBG) concentration or the binding ability of TBG, which may be affected by several drugs, including furosemide and heparin [16]. In contrast, FT3 and FT4 were not affected by these conditions. Thus, the FT3 level may be a better predictor of ICU mortality than other thyroid hormones, which is consistent with our findings.

Some limitations exist in our study. First, this was a retrospective study with a small sample size, which limited the statistical power. Therefore, additional patients need to be enrolled, and a prospective randomized multicenter study should be conducted. Second, patients with RACHS-1 scores of 5 or 6 are rare, which reduced the ability of the RACHS-1 to predict ICU mortality in children with CHD. Finally, in a retrospective study, it is difficult to collect data on perioperative and postoperative dopamine levels and steroid use; thus, we did not consider the relationship of thyroid hormones with dopamine and steroids, which may provide more robust evidence to assess the predominant predictor.

## Conclusions

The preoperative FT3 level may be a powerful and independent predictor of ICU mortality after CPB in infants with CHD who are younger than 3 months old.

## Data Availability

The datasets used in current study are available from the corresponding author on reasonable request.

## Abbreviations

ACC: Aortic Cross-Clamping
AUC: Area Under the Curve
CPB: Cardiopulmonary Bypass
CHD: Congenital Heart Disease
FT3: Free Triiodothyronine
FT4: Free Thyroxin
ICU: Intensive Care Unit
MV: Mechanical Ventilation
RACHS-1: Risk Adjustment in Congenital Heart Surgery-1
RCT: Randomized Controlled Trial
ROC: Receiver Operating Characteristic
TBG: Thyroxine-Binding Globulin
T3: Triiodothyronine
T4: Thyroxin
TT3: Total Triiodothyronine
TT4: Total Thyroxin
TSH: Thyroid Stimulating Hormone
